# Correction: Novel small molecule derivatives improve survivability in the cellular model of Huntington's disease *via* improving mitochondrial fusion

**DOI:** 10.1039/d5md90052b

**Published:** 2025-12-11

**Authors:** Pradeep Kodam, Vaishali Kumar, Paramita Pattanayak, Praharsh Vitta, Tanmay Chatterjee, Shuvadeep Maity

**Affiliations:** a Department of Biological Science, Birla Institute of Technology and Science, Pilani Hyderabad Campus, Jawahar Nagar, Kapra Mandal, Medchal District Telangana 500078 India shuvadeep@hyderabad.bits-pilani.ac.in; b Department of Chemistry, Birla Institute of Technology and Science, Pilani Hyderabad Campus, Jawahar Nagar, Kapra Mandal, Medchal District Telangana 500078 India tanmay@hyderabad.bits-pilani.ac.in

## Abstract

Correction for ‘Novel small molecule derivatives improve survivability in the cellular model of Huntington's disease *via* improving mitochondrial fusion’ by Pradeep Kodam *et al.*, *RSC Med. Chem.*, 2026, https://doi.org/10.1039/d5md00345h.

The authors regret that the initially uploaded supplementary information (SI) file contained modified ^1^H NMR spectra for the compounds **C-1** and **C-3**. The authors compared the spectra to the raw files and found the altered regions contained signals corresponding to grease (hydrocarbons, solvent residue) in both **C-1** and **C-3**, and a minor presence of another solvent, dichloromethane (DCM), in **C-3**. They state these are common residuals in organic synthesis and purification processes and do not compromise the purity or integrity of the compounds.

The authors have provided the raw data, which has been reviewed by an independent expert alongside the original images. They have verified that the data are consistent with the discussions and conclusions presented.

In addition, the authors found that the **C-5** HRMS spectrum was missing, and the **C-5** NMR spectrum had been inadvertently replaced in the initially published SI file.

The SI file has been updated with the missing HRMS spectrum of **C-5**, the correct ^1^H NMR spectrum of **C-5** and the unmodified NMR spectra of **C-1** & **C-3**.

The Royal Society of Chemistry apologises for these errors and any consequent inconvenience to authors and readers.

